# The diagnostic role of glycosaminoglycans in pleural effusions: A pilot study

**DOI:** 10.1186/1471-2466-9-9

**Published:** 2009-02-18

**Authors:** Rozina Vavetsi, Stefanos Bonovas, Paraskevi Polizou, Chrysanthi Papanastasopoulou, Georgia Dougekou, Nikolaos M Sitaras

**Affiliations:** 1 Department of Pharmacology, School of Medicine, University of Athens, Athens, Greece

## Abstract

**Background:**

Pleural effusions are classified into transudates and exudates. Various criteria have been used with Light's et al being the most accepted ones. Glycosaminoglycans (GAGs) have been detected during pleural fluids (PF) analysis in various causes. In this pilot study, we investigated: (a) the usefulness of GAGs in the assessment of pleural effusions, and (b) whether and in what way GAGs correlate with established criteria used to indicate an exudate.

**Methods:**

LDH, total protein, cholesterol and GAG levels were measured in pleural fluid and serum from 50 patients with pleural effusion. GAG levels were defined by the photometric method of Hata. The discriminative properties of pleural GAGs (pGAG), pleural fluid/serum GAG ratio (GAGR), serum GAGs (sGAG) and serum LDH (sLDH) were explored with ROC analysis.

**Results:**

According to ROC analysis, pGAG and GAGR exhibited satisfactory discriminative properties in the separation of pleural effusions. For GAGR, at a 1.1 cut off point, sensitivity and specificity reached 75.6%; 95%CI: 60.5–87.1 and 100%; 95%CI: 47.8–100, respectively. For pGAG at a cut off value of 8.4 μg/ml, these percentages changed to 86.7%; 95%CI: 73.2–94.9 and 100%; 95%CI: 47.8–100. The study also revealed the differential role of sGAG between malignancies and benign cases, scoring 68.8%; 95%CI: 50.0–83.9 for sensitivity, and 84.6%; 95%CI: 54.5–97.6 for specificity at a 7.8 μg/ml cut off.

**Conclusion:**

Our results suggest that glycosaminoglycan measurement of both serum and pleural effusions could be useful for simultaneous differentiation of exudates from transudates, and of malignant from benign exudates.

## Background

Pleural effusions, being a common medical problem, have been classically divided into transudates and exudates. Differentiation is of particular importance because in the case of a transudate, aetiology and therapy are directed to the underlying congestive heart failure, cirrhosis, or nephrosis. Alternatively, if the effusion proves to be an exudate, malignancy is suspected and a more extensive diagnostic procedure is needed. According to Light's criteria [1,2] which still remain the most accurate ones, pleural fluid is an exudate if any of the following are met: (a) pleural fluid to serum ratio of total protein > 0.5, (b) pleural fluid to serum ratio of lactic acid dehydrogenase (LDH) > 0.6, and (c) pleural fluid LDH > 3/4 the upper limit of normal serum LDH. Additional criteria-such as pleural fluid cholesterol level > 60 mg/dL or > 45 mg/d [3,4], and a serum fluid albumin gradient > 1.2 mg/dl [5] – have been also proposed in parallel or in combination with Light to optimize sensitivity and specificity of diagnosis. Criteria, however, regarding the differentiation between malignant and benign exudates have not been yet established.

Glycosaminoglycans (GAGs) are long, straight chain polysaccharides composed of repeated disaccharide units that are mainly attached to a core protein to form proteoglycans. GAGs are subdivided into the chondroitin sulfates (chondroitin-4-sulfate and chondroitin-6-sulfate), heparan sulfates (heparan sulfate and heparin), dermatan sulfate, keratan sulfate and hyaluronic acid and their presence has been studied in several deceases. They are normally produced by the mesothelial cells of pleural cavity and large amounts of them, mainly hyaluronic acid and chondroitin sulphate [6,7] have already been reported in pleural effusions. Afify et al [8] concluded that hyaluronan and its cell surface receptor CD44v6 can serve as an ancillary test to cytological examination, to distinguish between malignant and benign effusions. Welker et al [9] studied the combined use of cytology and hyaluronic acid analysis in improving the detection of malignant mesothelioma in pleural effusions, reporting impressive results.

In this pilot study we investigated: (a) the usefulness of GAGs in the assessment of pleural effusions, and (b) whether and in what way GAGs correlate with established criteria used to indicate an exudate.

## Methods

### Patients

All the patients of the study were referred to the Respiratory Unit at 'Sotiria' General Hospital, Athens, Greece, for a prospective investigation and removal of pleural effusion. None of the patients followed previous diuretic diet. During the study period, pleural effusion samples from 50 patients were collected, 29 men and 21 women, mean age 65.4 ± 15.9 years. Pleural fluid and serum samples were collected from all patients centrifuged and kept at -70°C immediately. All patients were followed for at least 3 months, or until a final cause of the pleural fluid was determined (Table 1). Effusions were determined as exudates or transudates according to the criteria of Light. Malignancies were established by positive cytology in combination with pleural fluid differential cell counts and immunocytochemistry techniques when needed. Pleural fluid positive cell cultures followed by positive stains verified tuberculosis and parapneumonic effusions. Amylase measurement was requested to confirm pancreatitis. All transudates were diagnosed as cardiac failures. Evaluation was based on standard clinical procedures (NYHA stage 3 and 4 and/or cardiography injection fraction < 30). Thoracoscopy and pleural biopsy were performed in cases of undiagnosed exudates. The study was conducted in accordance with the ethical principles set forth in the Declaration of Helsinki and with local regulations. The protocol was approved by the Institutional Ethics Committee of the School of Medicine, University of Athens, reference number 1575/96.

**Table 1:** Clinical diagnosis of patients' pleural effusions.

**Diagnosis**	**n**
**Exudates (n = 45)**	
Malignancies	32
Parapneumonic	1
Tuberculosis	1
Pleuritis	4
Miscellaneous*	7
**Transudates (n = 5)**	
Cardiac failure	5
Total	50

* Polyartiritis nodosa 1, collagenous disease 1, pancreatitis 1, post-traumatic pleuritis 1, eosinophilic pleuritis 2, acute myelogenic leukaemia 1.

### Glycosaminoglycan measurement

All samples were analysed for GAGs according to Hata et al [10]. Briefly, 1 ml of each sample was dehydrated by adding 10 ml chilled acetone. The mixture was stirred for 30 min at room temperature and centrifuged at 1500 rpm for 15 min at 4°C. The dehydrated sample was delipidated by washing twice with 10 ml of ether and the precipitate was air-dried. The defatted dry powder was resuspended in 1 ml of 0.5 M chilled NaOH and stirred at 4°C overnight. Then it was neutralised with 1 M HCl to pH 6–8, an equal volume of 0.1 M Tris-HCl buffer was added and the sample was heated for 30 min in a boiling bath. The sample solution was digested with pronase E in two doses of 1 mg at 24 h intervals, at 50°C. After being acidified with 1 M HCl to pH 5–6, 2 ml cetylpyridinium chloride (CPC) were added and kept at 4°C overnight. The precipitate was collected by centrifugation at 4,000 rpm for 30 min at 4°C and then washed twice with 5 ml of 98% ethanol containing 10% CH_3_COOK. The final precipitate was dissolved in 1 ml of 0.05 Tris-HCl buffer. Uronic acid was measured using the uronic acid-carbazole reaction and GAGs were expressed as μg uronic acid per ml of pleural fluid or serum [11]. Chemicals were purchased by Sigma (S. Louis, Missouri).

### Measurement of biochemical markers

Adequate amounts of the samples were kept to analyze for glucose, total protein, LDH, cholesterol (CHOL) and triglycerides (TGs) in pleural effusions and sera. All measurements were performed in Olympus AU-640 analyser (Medicon), at the biochemistry laboratory of the 'Sotiria' General Hospital.

### Statistical analysis

Analysis of results was performed with two statistical packages: the SPSS 10 and the MedCalc software. Medians with the first and third quartiles were calculated for overall nine parameters in all four study groups, i.e. transudates, exudates, malignancies and benign cases. Because most variables were not normally distributed, variables were compared by means of non-parametric tests. Differences among the three groups (malignancies, benign and transudates) were assessed with the Kruskal Wallis equality of populations rank test for three independent samples, followed by the Wilcoxon rank-sum (Mann-Whitney) test. Correlations were performed with Spearman's rank order coefficient. The discriminative properties of pGAG, GAGR, sGAG and serum LDH (sLDH) were explored with ROC analysis. All p-values < 0.05 were considered statistically significant.

## Results

### Malignancies vs. benign vs. transudates

Patients were classified into three groups. Group A included 32 patients with malignant pleural effusions. Group B consisted of 13 patients with benign disease, and group C included 5 transudate effusions. Medians with the first and third quartiles for each of the nine parameters analyzed in the three studied groups are shown in Table 2. All other parameters except sGAG, sTP and sLDH were significantly higher in malignancies and benign disease than transudate effusions. sGAG and sLDH were both significantly increased in malignancies compared to benign cases (p = 0.002, and p = 0.021, respectively).

**Table 2:** Medians with the first and third quartiles in parentheses and ranges in brackets of the nine parameters, in the malignant (A), benign (B) and transudates (C) group, respectively.

	**malignant (A), (n = 32)**	**benign (B), (n = 13)**	**transudates (C), (n = 5)**	***P*_total_**	***P***
**pGAG**	14.0 (11.0–20.7), [4.0–52.6]	15.9 (9.3–23.7), [5.9–59.7]	4.9 (3.9–7.2), [3.3–8.4]	0.004	AvsB p = 0.582; AvsC p < 0.001; BvsC p = 0.001

**sGAG**	8.7 (7.2–11.2), [2.3–23.0]	6.6 (5.1–7.7), [3.3–9.9]	8.6 (6.6–13.0), [5.5–16.4]	0.007	AvsB p = 0.002; AvsC p = 0.983; BvsC p = 0.059

**GAGR**	1.7 (1.1–2.5), [0.5–4.3]	2.3 (1.3–5.0), [0.9–8.6]	0.5 (0.3–1.1), [0.3–1.1]	0.003	AvsB p = 0.059; AvsC p = 0.004; BvsC p = 0.001

**pTP**	4.2 (3.4–5.2), [1.6–6.1]	4.3 (3.6–4.8), [3.0–5.2]	2.6 (2.1–2.7), [1.6–2.8]	0.003	AvsB p = 0.930; AvsC p = 0.001; BvsC p < 0.001

**sTP**	6.3 (5.7–7.2), [4.7–8.6]	6.6 (6.1–7.1), [5.3–7.9]	6.3 (5.0–6.7), [4.8–7.0]	0.444	AvsB p = 0.607; AvsC p = 0.350; BvsC p = 0.173

**TPR**	0.6 (0.6–0.7), [0.3–2.0]	0.6 (0.6–0.7), [0.5–2.0]	0.4 (0.3–0.5), [0.2–0.5]	0.009	AvsB p = 0.920; AvsC p = 0.002; BvsC p = 0.001

**pLDH**	333.5 (207.0–666.2), [74–7000]	242.0 (144.0–521.0), [73–677]	103.0 (66.5–144.5), [63–177]	0.004	AvsB p = 0.215; AvsC p < 0.001; BvsC p = 0.014

**sLDH**	256.0 (204.2–366.0), [163–1018]	201.0 (165.5–253.0), [129–309]	262.0 (237.0–267.5), [214–272]	0.056	AvsB p = 0.210; AvsC p = 0.914; BvsC p = 0.117

**LDHR**	1.2 (0.8–1.8), [0.3–9.5]	1.4 (0.6–2.8), [0.4–3.2]	0.4 (0.3–1.00), [0.2–0.7]	0.008	AvsB p = 0.773; AvsC p = 0.001; BvsC p = 0.004

Column 4 presents total scores of *p*-values as these were formulated by the Kruskal-Wallis rank test. Differences among pairs are included in the last column.

We further assessed sGAG and sLDH as discriminative markers in malignant vs. benign disease. The best cut off points was selected using ROC analysis. With a 7.8 μg/ml cut off value, sGAG showed sensitivity 68.7%; 95%C.I: 50.0–83.9 and specificity 84.6%; 95%C.I: 54.5–97.6. For sLDH and a 201 u/l cut off, these percentages scored 81.2%; 95%C.I: 63.6–92.7 and 53.8%; 95%C.I: 25.2–80.7, respectively (Table 3).

**Table 3:** Sensitivity, specificity, and 95% CI, for the cut off values of sGAG and sLDH obtained with ROC analysis, between malignant and benign exudates.

	**Cut off point**	**Sensitivity (%)**	**95% CI**	**Specificity (%)**	**95% CI**
**sGAG**	7.8 μg/ml	68.7	50.0–83.9	84.6	54.5–97.6

**sLDH**	201 u/l	81.2	63.6–92.7	53.8	25.2–80.7

### Exudates vs. transudates

Differences among transudates vs. all exudates, numbering 5 and 45 patients respectively, were also studied. Pleural fluid GAGs (pGAG) and the pleural fluid/serum GAG ratio (GAGR) were both significantly higher (p < 0.001, p = 0.001) in the exudate group (Table 4). This was also observed with pleural fluid total protein, pLDH, and LDHR (p < 0.001 for all three parameters). No statistical difference was found in serum GAGs (sGAG); on the contrary values were very close. According to ROC analysis, both GAGR and pGAG exhibited satisfactory discriminative properties. The 1.1 cut off value demonstrated the highest accuracy for GAGR (sensitivity 75.6%; 95%CI: 60.5–87.1; specificity 100%; 95%CI: 47.8–100), whereas a cut off point of 8.4 μg/ml yielded optimum results for pGAG (sensitivity 86.7%; 95%CI: 73.2–94.9; specificity 100%; 95%CI: 47.8–100). Sensitivities and specificities of Light's criteria were also estimated (Table 5).

**Table 4:** Medians with the first and third quartiles in parentheses, and ranges in brackets, of the nine parameters, in the exudate and transudate group, respectively.

	**Exudates (n = 45)**	**Transudates (n = 5)**	**p**
**pGAG**	14.4 (10.4–21.3), [4.0–59.7]	4.9 (3.9–7.2), [3.3–8.4]	< 0.001

**sGAG**	8.0 (6.6–9.8), [2.3–23.0]	8.6 (6.6–13.0), [5.5–16.4]	0.571

**GAGR**	1.9 (1.1–2.9), [0.5–8.6]	0.5 (0.3–1.1), [0.3–1.1]	0.001

**pTP**	4.2 (3.4–5.0), [1.6–6.1]	2.6 (2.1–2.7), [1.6–2.8]	< 0.001

**sTP**	6.5 (5.8–7.1), [4.7–8.6]	6.3 (5.0–6.7), [4.8–7.0]	0.267

**TPR**	0.6 (0.6–0.7), [0.3–2.0]	0.4 (0.3–0.5), [0.2–0.5]	0.001

**pLDH**	331.0 (195.0–596.0), [73–7000]	103.0 (66.5–144.5), [63–177]	< 0.001

**sLDH**	225.0 (200.5–324.5), [129–1018]	262.0 (237.0–267.5), [214–272]	0.550

**LDHR**	1.3 (0.7–2.0), [0.3–9.5]	0.4 (0.3–1.0), [0.2–0.7]	< 0.001

For each parameter, comparisons between the two groups were made to establish statistically significant differences.

**Table 5:** Sensitivity, specificity, and 95% CI, for the cut off values of TPR, LDHR, LDH, GAGR, and pGAG, obtained by ROC analysis between exudate and transudate effusions.

	**Cut off point**	**Sensitivity (%)**	**95% CI**	**Specificity (%)**	**95% CI**
**TPR**	0.5	93.9	81.7–98.6	60.0	14.7–94.7

**LDHR**	0.6	84.4	70.5–93.5	80.0	28.4–99.5

**LDH**	250 u/l	88.9	75.9–96.3	80.0	28.4–99.5

**GAGR**	1.1	75.6	60.5–87.1	100	47.8–100

**pGAG**	8.4 μg/ml	86.7	73.2–94.9	100	47.8–100

### Correlations

**Figure 1:**
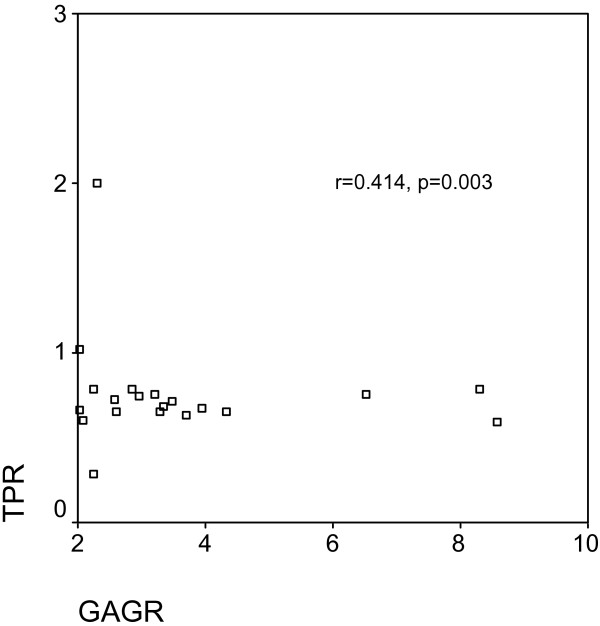
**GAGR is positively correlated with TPR**.

**Figure 2:**
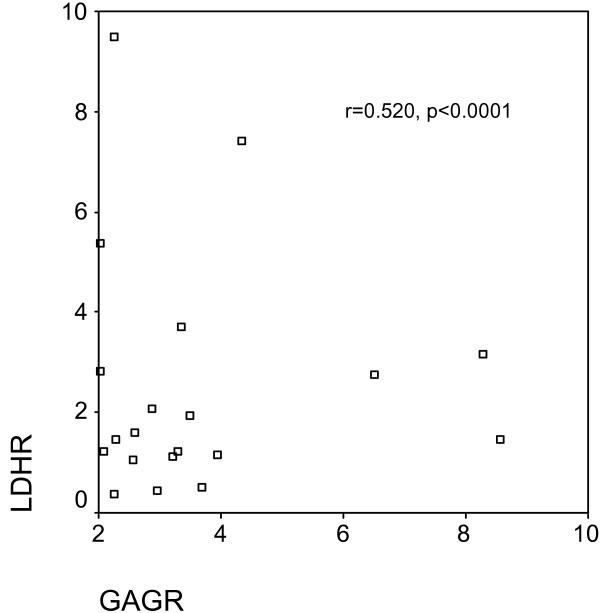
**GAGR is positively correlated with LDHR**.

**Figure 3:**
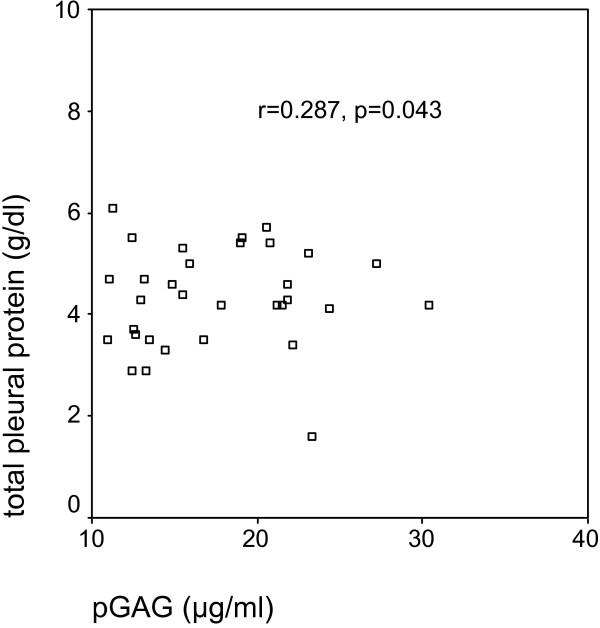
**pGAR levels are positively correlated with total pleural protein**.

**Figure 4:**
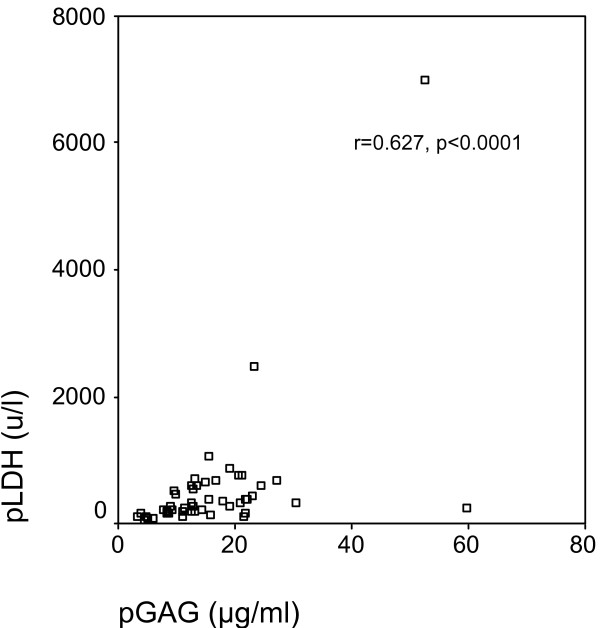
**pGAG levels are positively correlated with pLDH**.

**Figure 5:**
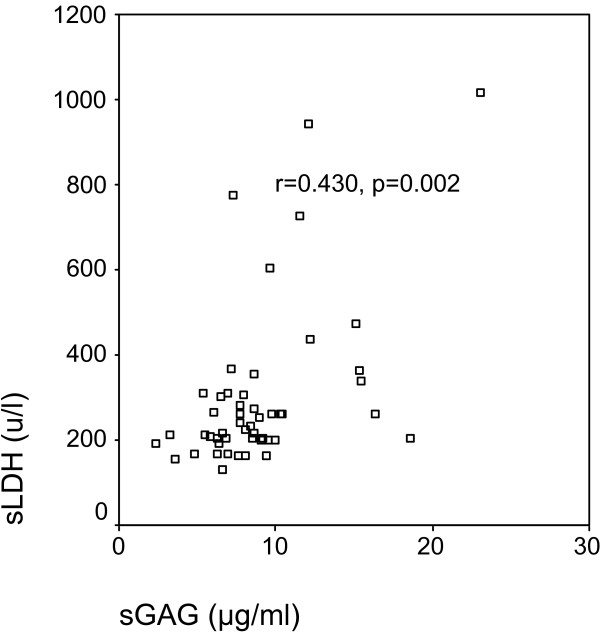
**sGAG levels are positively correlated with sLDH**.

In the total group of 50 patients, GAGR correlated significantly with TPR (r = 0.414, p = 0.003, Figure 1), and LDHR (r = 0.520, p < 0.0001, Figure 2). pGAG levels exhibited a mild positive correlation with total pleural protein (r = 0.287, p = 0.043, Figure 3), and a strong positive correlation with pLDH (r = 0.627, p < 0.0001, Figure 4). sGAG and sLDH were also positively correlated (r = 0.430, p = 0.002, Figure 5). No statistically significant correlation was observed between total serum protein with either sGAG, or sLDH.

## Discussion

This study investigates the discriminative role of glycosaminoglycans between (a) exudates and transudates, and (b) malignant and benign exudates, using ROC analysis. The statistically significant raise of pGAG in the exudate group indicate the possible existence of pathological processes that result in either increased permeability, local production or decreased clearance of the pleural glycosaminoglycans similar to those governing the protein and LDH content. This suggestion is further supported by the fact that GAGR correlate with TPR and LDHR, while pGAG correlate with total pleural protein and pLDH. Interestingly, pGAG levels showed no difference between malignant and benign cases, with sGAG, however, showing a significant increase in malignancies.

Since 1972, when Light first published his criteria for the differentiation of pleural effusions, additional markers have been proposed. Cholesterol, bilirubin and albumin gradient have been extensively studied. According to a meta-analysis by Heffner et al [12], the most frequently used markers, except bilirubin, perform well, although the protein ratio appears marginally the best. In our study, both pGAG and GAGR exhibited interesting discriminating properties regarding transudates vs. exudates, with pGAG giving slightly higher sensitivity scores. Although the number of patients in the transudate group was very small (n = 5), both GAGR and pGAG correctly detected them all, maximizing specificity, while pGAG misclassified 6 out of the 45 exudates. GAGR, however, exhibited the poorest sensitivity over the other four parameters (Table 4). TPR correctly classified 42 of the 45 exudates, giving the highest sensitivity (93.3%), but in expense of specificity (60%). LDH and pGAG performed best compared to the other criteria. Among benign vs. malignant cases, sGAG was the marker with relatively good sensitivity and specificity scores. sLDH -a parameter classically used in Light's criteria-, gave a higher sensitivity percentage compared to sGAG, followed however by a low specificity one (Table 5).

Because what causes a transudate is usually easily detected, researchers have been mainly focused on the diagnosis of exudates. Especially, the establishment of malignant effusions continues to be a clinical problem as its final diagnosis depends heavily on cytological examination, a technique with low sensitivity scores. However, in every day clinical practice transudates are more common and if the cause is not that clear they as well call for a precise and reliable diagnosis. A marker that would direct initial clinical judgment on either transudates or malignancies could be a valuable aid. Though our study is limited by the small sample size and the re-testing of reliability in the same sample, findings indicate that GAGs could be possible candidates for such a role under the condition that results are further tested and justified over a much larger number of patients.

## Conclusion

Although many studies appear in the literature to investigate the significance of glycosaminoglycans' presence in pleural effusions, only few studies have been concentrated in the identification of markers that can differentiate between malignant and benign disease. The fact that both benign and malignant effusions share the same lymphocytic exudative profile makes it difficult to determine a specific diagnosis and the need for establishing specific criteria is growing. Investigations include markers such as hyaluronan, interleukin-6, tumor necrosis factor, and C-reactive protein. Bernard et al [13] also proposed the use of F-FDG PET imaging for the differentiation between benign and malignant exudates, succeeding impressive results. Our results suggest that glycosaminoglycan measurement of both serum and pleural effusions could be useful for simultaneous differentiation of exudates from transudates and of malignant from benign exudates. These findings guarantee the need for further investigation.

## Abbreviations

GAGs: glycosaminoglycans; pGAG: pleural glycosaminoglycans; sGAG: serum glycosaminoglycans; GAGR: pleural fluid to serum ratio of glycosaminoglycans; pLDH: pleural fluid lactic acid dehydrogenase; sLDH: serum lactic acid dehydrogenase; LDHR: pleural fluid to serum ratio of lactic acid dehydrogenase; TPR: pleural fluid to serum ratio of total protein.

## Competing interests

The authors declare that they have no competing interests.

## Authors' contributions

RV participated in the design of the study, performed statistical analysis and drafted the manuscript. SB performed ROC analysis. PP collected the samples and measured the biochemical markers of the study. CP and GD carried out the glycosaminoglycan measurements. NS conceived of the study, participated in its design and coordination, and helped to draft the manuscript. All authors read and approved the final manuscript.

## Pre-publication history

The pre-publication history for this paper can be accessed here:


